# Stratigraphic and structural architecture of the inner ramp carbonates in the Northern Galala Plateau, Egypt: synergizing remote sensing and field data

**DOI:** 10.1038/s41598-026-35896-6

**Published:** 2026-02-05

**Authors:** Mohamed S. Fathy, Mohamed A. Abd El‑Wahed, Mahmoud Faris, Abdallah S. Ali, Mohamed Attia

**Affiliations:** 1 Geology Department, Faculty of Science, Tanta University, P.O. Box: 31527, Tanta, Egypt; 2 State Key Laboratory of Continental Evolution and Early Life, Department of Geology, Northwest University, Xi’an, 710069 China; 3 Geology Department, Faculty of Science, Kafr El Sheikh University, P.O. Box: 33511, Kafr El Sheikh, Egypt

**Keywords:** Northern Galala Plateau, Inner ramp carbonates, Early paleogene, Southern Galala formation, Egypt, Environmental sciences, Solid Earth sciences

## Abstract

The new high-altitude road in New Galala City offers a valuable opportunity to study the carbonate platform of the Southern Galala Formation at the Northern Galala Plateau in Egypt. The research examines this carbonate platform through remote sensing, structural, and stratigraphic methods. For the first time, remote sensing techniques using Landsat-9 have been applied to differentiate the carbonate platform rocks and their depositional environments. Tectonic uplift has shaped a complex topography and high structural elevation, resulting in a rimmed platform with varying slope angles. Lithostratigraphically, the Southern Galala Formation has been divided into three new formal members: Wadi Al-Rasis, Gebel Ealyan, and New Galala City. The Wadi Al-Rasis and New Galala City members are mainly composed of pale brown, thin, laminated dolostones with some siliciclastics. These members are characterized by microbial mudstone and wackestone microfacies of tidal flat environments. The Gebel Ealyan Member features grey, fossil-rich limestones with sediments that have undergone karstification. Key fossils include large benthonic foraminifera. Microscopic studies of this member reveal various bioclastic packstone/grainstone microfacies of lagoon and shoals’ environments. Critical diagenetic processes include micritization, cementation, and dolomitization, thereby enhancing the economic significance of the rocks as hosts to hydrocarbon reserves, groundwater aquifers, and industrial minerals. Tectonic uplift, eustatic sea-level changes, and sedimentary dynamics influence the structures that control the studied inner-ramp carbonates.

## Introduction

A carbonate platform is a substantial, underwater calcium carbonate (CaCO₃) build-up in a shallow marine setting, mainly formed by the biological activity of carbonate-secreting organisms. Standard models, such as the carbonate ramp (gently sloping) and rimmed shelf (with a clear break in slope), divide the platform into several distinct depositional zones based on energy, water depth, and circulation. These deposits are vulnerable to shifts in climate, sea level, and water chemistry, leading to various facies including tidal flats, lagoons, and protected bays^1–4^. The three primary zones are: outer platform (high-energy, wave-disturbed environment with reefs and/or sand shoals); platform interior (lower-energy zone); and inner platform (the most landward part of the platform interior, directly next to the shoreline and most affected by subaerial exposure).

The inner platform environment features shallow water depths (0–20 m), restricted circulation, low to moderate energy levels (but influenced by tides and storms), and variable salinity ranging from brackish to hyper-salinity. Due to its extremely shallow nature, this area is highly vulnerable to sea-level changes, leading to frequent subaerial exposure and the formation of exposure features such as paleosols and karst. Nonetheless, the main factor controlling inner platform facies is climate (arid versus humid), which determines salinity and influences biotic communities and early diagenetic processes^5^.

Facies association of the inner platform subdivided from land to basin into tidal flat, restricted lagoons, and shoals^3,5,6^. The restricted lagoons and bays are characterized by peloidal and skeletal lime mudstone and wackestone. Skeletal grains are typically from stress-tolerant organisms: miliolids, foraminifers, ostracods, thin-shelled bivalves, and green algae. Laminated microbial mats (stromatolites), mud-cracks, evaporite, and fenestral (bird’s eye) structure with burrowed peloidal wackestone/packstone are the main facies of the tidal flats. On the other hand, the sand shoals and islands are discriminated by well-sorted grainstone composed of ooids, peloids, and skeletal debris (e.g., gastropods, foraminifera, algae).

Carbonate rocks are predominantly biogenic and highly responsive to changes in their physicochemical environment^7–10^. Diagenesis encompasses all physical, chemical, and biological processes that alter sediments after deposition and before metamorphism. However, diagenesis in inner-platform carbonates occurs across a series of environments, each with distinct processes. Cementation: precipitation of carbonate cement from seawater, primarily as aragonite and/or calcite^9^. Dissolution: meteoric water readily dissolves unstable minerals, particularly aragonite. This creates secondary porosity, including moldic and vuggy porosity. Dolomitization: occurs via seepage reflux or from modified seawater. It can enhance porosity by creating intercrystalline pores without cementation^5,6,10^.

The economic importance of the inner ramp carbonate is disproportionately high relative to their volume due to a combination of primary depositional features and, most importantly, extensive secondary diagenetic modification^11–14^. This diagenesis occurs in a unique geochemical environment characterized by frequent sea-level changes and climate, which create conditions for pervasive mineral alteration and porosity development. The inner platform carbonates constitute many of the world’s most prolific hydrocarbon reservoirs. Their value stems from their ability to store (porosity) and produce (permeability) oil and gas. Grainstone shoals possess excellent primary interparticle porosity and permeability. Fenestral porosity of “bird’s eye” structure forms from gas bubbles trapped in microbial mats or from the decay of organic material. This provides significant, well-connected porosity in otherwise muddy rock. The dolomitization process causes a net volume reduction, creating abundant intercrystalline porosity. This creates a tough, porous rock with a sugar-like texture ideal for fluid flow^5^. The Upper Jurassic Arab Formation of Saudi Arabia is a classic example of dolomitized inner-platform facies that form reservoirs for some of the world’s largest oil and gas fields^15^ (e.g., Ghawar Field). Additionally, these rocks form a good aquifer in Wadi Araba, south of the studied plateau (Salem et al., 2024). During periods of sea-level fall, the inner platform is exposed to meteoric (rain) water^16,17^. This slightly acidic water dissolves carbonate rock, creating a complex network of secondary porosity. Features include vugs, molds, enlarged fractures, and extensive cave systems^18^. This dramatically increases porosity and permeability, building highly productive “paleokarst” reservoirs. Many carbonate reservoirs have a significant karstic component, which is a key factor in their exemplary performance.

The use of remote sensing data and image processing techniques can provide detailed information for the identification of lithological features, delineation of geological structures, and investigation of hydrothermal alteration in certain ore deposits within the Eastern Desert of Egypt^19–29^. In geological applications using remote sensing data, lithological and structural mapping remains one of the most significant subjects, with several advancements made through the lithological, structural, and alteration zones mapping^21,24,25,27–29^. Accurate lithological and structural mapping is essential for addressing tectonic evolution and mineral exploration in any area^30–34^. To enhance the geological and structural map of the specified region, we integrated multisensor data, including Landsat-9 and Sentinel-1, with field data and petrographic analysis to achieve our objectives.

## Geological setting

**Fig. 1:**
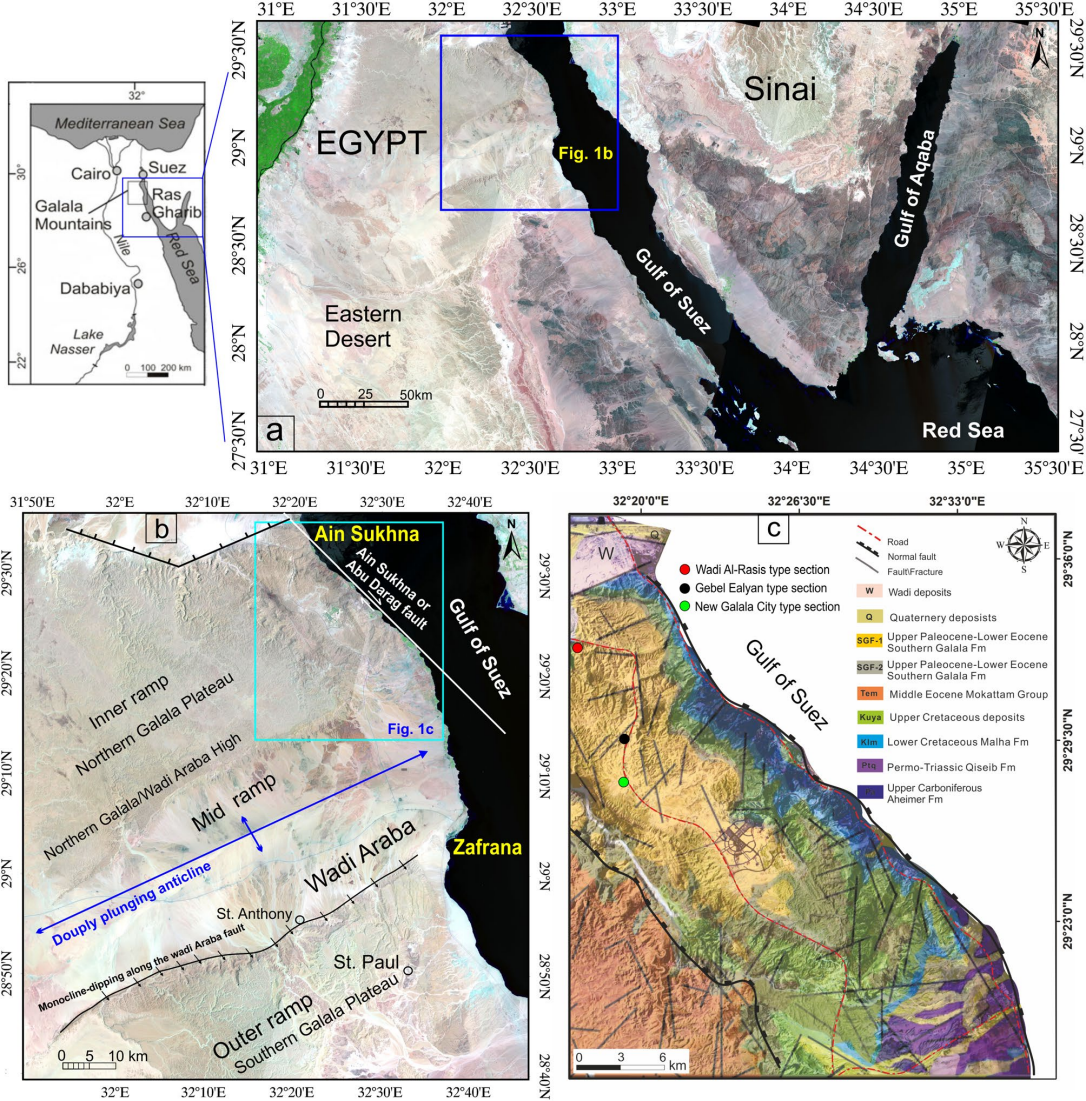
(**a**) Landsat image showing the Location of the studied Northern Galal Plateau, Egypt. (**b**) Landsat image showing tectono-topographic and carbonate platform features of the Northern Galala Plateau (modified after Höntzsch et al.^36^; Farouk^54^. (**c**) Geological map of the studied area (modified after Conoco^44^. Location (1) is the type locality of the Wadi Rasis Member, (2) type locality of the Gebel Ealyan Member, (3) type locality of the New Galala City Member. These figures were created and processed by ENVI v. 5.6.2. software: https://www.l3harrisgeospatial.com/Software-Technology/ENVI), mainly used for image processing, and ArcGIS Desktop 10.8. (https://www.esri.com/en-us/arcgis/products/arcgis-desktop/overview/).

The Northern Galala Plateau is one of the most extensive carbonate tablelands in the Eastern Desert of Egypt. It is situated directly north of the Wadi Araba depression, west of the Gulf of Suez, and east of Cairo (Fig. 1a), making it a region of growing strategic and economic importance. The Egyptian government is building several new cities and major tourist resorts on the plateau (most notably New Galala City), capitalizing on its stunning views, mild climate, and proximity to Cairo and the Red Sea coast.

The stratigraphic succession of the Northern Galala Plateau begins with Precambrian crystalline rocks at the base, followed by the Phanerozoic Era. The Phanerozoic consists mainly of Cambrian-Lower Cretaceous siliciclastic-dominated rocks succeeded by Upper Cretaceous-Eocene carbonate platform^35^.

The formation of the Galala carbonate platform during the Early Paleogene was significantly influenced by syndepositional tectonic activities associated with the Wadi Araba Fault (Fig. 1b), which is part of the Syrian Arc System^36–38^. Three separate tectono-sedimentary phases of the platform, ranging from the Paleocene-Eocene Thermal Maximum (PETM) to the latest Early Eocene, were identified, with the end of tectonic activity signified by the absence of siliciclastic deposits^36^. Scheibner et al.^39^ delineated five stages of the platform with respect to progradation and retrogradation of the platform margin, which illustrate the evolution of the study area from the Maastrichtian to the earliest Eocene (stages A–E).

**Fig. 2:**
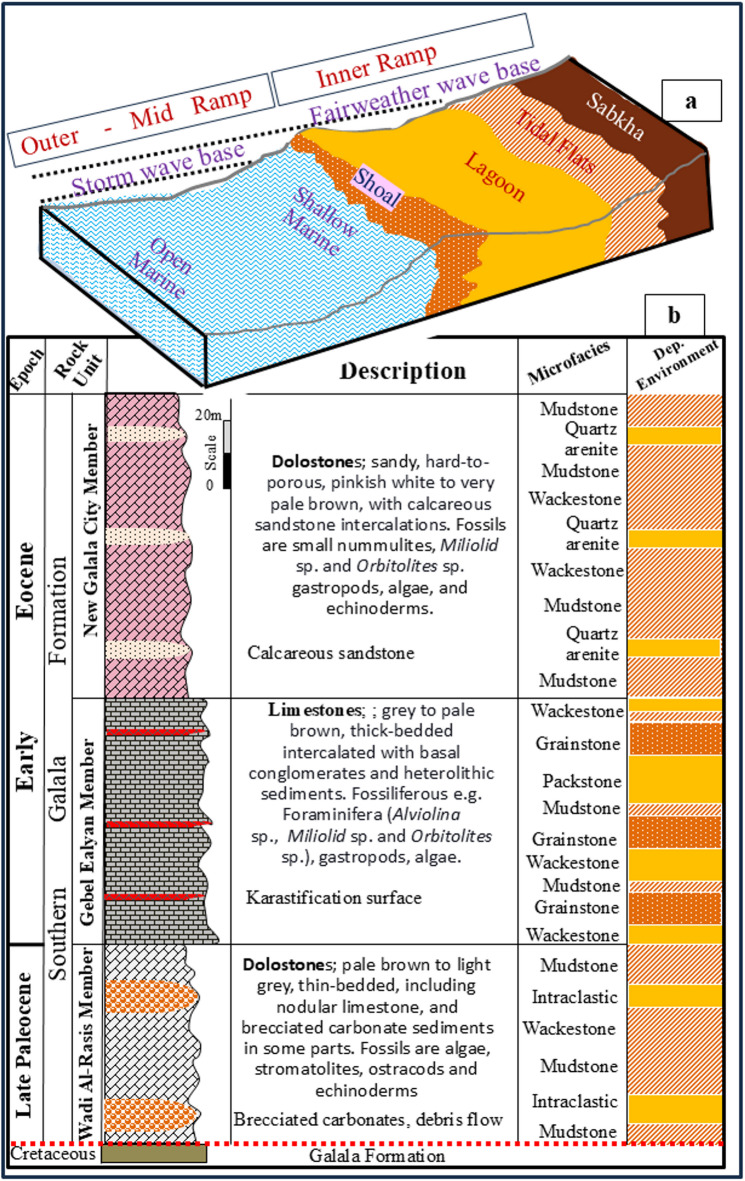
(**a**) The sketch figure shows the inner ramp platform’s sub-environments (sabkha, tidal flat, lagoon, and shoal). (**b**) Lithostratigraphic column, microfacies types, and depositional environments of the Upper Paleocene- Lower Eocene Southern Galala Formation in the Northern Galala Plateau, Egypt.

The Northern Galala Plateau is located approximately 800 km north of the African craton and has been influenced by tectonic uplift along the Syrian Arc fold belt. Such uplift has created diverse sedimentary environments, including inner ramp, mid-ramp, and outer ramp settings (Figs. 1b and 2a), each distinguished by unique lithological and biological characteristics^36,41^.

The inner ramp environment of the plateau is particularly remarkable for its shallow marine deposits. These deposits predominantly consist of bioclastic and oolitic grainstones, indicating high-energy conditions typical of shallow-marine settings. The inner ramp is affected by wave and tidal forces, accumulating coarser materials in channels and carbonate muds on tidal flats. The sedimentary structures frequently exhibit decimetre to metre-scale cross-bedding and horizontal stratification, which reflect the dynamic processes occurring in this environment^36–38^.

The Lower Eocene succession in the Galala Mountains (Figs. 1c and 2b) is distinguished by three prominent lithostratigraphic units corresponding to diverse depositional environments ranging from inner-platform to basin settings^39,40,42,43^. The Esna Formation, representing the uppermost Paleocene to Lower Eocene, primarily consists of basinal marls and shales, with an average palaeo-depth of 200 m and minimal influence from shallow marine conditions. Subsequently, the Thebes Formation is recognized for its deep-water facies, characterized by cherts alongside alternating chalky marls and limestones. This formation signifies the initial occurrence of chalky marls within the succession^39,42,43^. The geological map shows these successions changed northward to form the Middle Eocene Mokattam Group^44^. Finally, the Southern Galala Formation (Fig. 2) is characterized by shallow-marine limestones, dolostones, and conglomerates and shows distinct facies similarities with the Thebes Formation, especially in mid-ramp regions^39^. The present-day sedimentary successions indicate a history of sea-level fluctuations and tectonic movements associated with the Syrian Arc System^36,45^.

## Materials and methods

### Satellite dataset

Landsat-9 utilizes the Operational Land Imager 2 (OLI-2) to observe changes on Earth, capturing images across the visible, near-infrared (VNIR), and shortwave-infrared spectrum (SWIR) with a pixel resolution of 30 m, along with a high-resolution panchromatic band (Band 8) at 15 m, as specified in Table 1. Additionally, Landsat-9 is equipped with two thermal infrared (TIR) bands with a 100 m spatial resolution, provided by the TIR-2 sensor. A terrain-corrected, cloud-free Landsat 9 scene (LC09_L2SP_176040_20240809_20240810_02_T1) exhibiting surface reflectance was used as optical satellite data to investigate the lithological characteristics within the research area.

**Table 1:** The prominent characteristics of the Landsat-9.

Sensor	Band Number	Spectral Region	Spectral range (µm)	Resolution (m)	Swath width (km)
Landsat- 9	B8: Panchromatic	VNIR	0.503–0.676	15	185
	B1: Coastal aerosol		0.433–0.453	30	
	B2: Blue		0.450–0.515		
	B3: Green		0.525–0.600		
	B4: Red		0.630–0.680		
	B5: NIR		0.845–0.885		
	B6: SWIR1	SWIR	1.560–1.660		
	B7: SWIR2		2.100–2.300		
	B9: Cirrus		1.363–1.384		
	B10: TIRS	TIR	10.60–11.19	100	
	B11: TIRS		11.50–12.51		

For radar data, the automatic extraction of lineaments and their domain trends was implemented using the LINE extraction approach supported by PCI-Geomatica and RockWork software, which was applied on Sentinel-1(S1B_IW_GRDH_1SDV_20211216T034418_20211216T034443_030045_039658_B231.SAFE) dual polarization (HV, VV Table 2) radar data in the study area. Both the optical and radar data were layered, stacked, and subsetted to highlight the area of interest, and they were projected to the WGS-84 UTM Zone 36 N coordinate system.

**Table 2:** The emphasized features of the Sentinel-1 radar data.

AM	SM	IW	EW	WV
Beam Mode	S1 to S6	IW1 to IW3	EW1to EW5	WV1&WV2
Center Frequency	C-band (5.405 GHz)			
Polarization	SP (HH or VV) DP (HH + HV and VV + VH)			
Spatial resolution (range x azimuth) (m)	5 × 5	5 × 20	25 × 100	5 × 20
Band width (Km)	80	250	4	20 × 20
Chirp bandwidth (MHz)	87.6–42.2	56.5–42.8	22.2–10.4	74.5 & 48.2
Incidence angle (deg)	20–43°	30–42°	20–44°	23 & 36.5°

Acquisition mode “AM”, Stripmap “SM”, Interferometric wide swath “IW”, Extra wide swath “EW”, Wave “WV”, Single Polarization “SP”, Daul Polarization “DP”, Degree “deg”.

The imagery from Landsat-9 has been atmospherically corrected as a preprocessing step using the Internal Average Relative Reflection (IARR) method in Envi to reduce or eliminate atmospheric effects. At the same time, the enhanced Lee filter was applied to the VH and VV polarizations of the radar data to reduce speckle in the S1B imagery while preserving texture information. The image processing techniques applied to Landsat-9, including False Color Composite (FCC), Band Ratios (BRs), and Minimum Noise Fraction, were used to identify lithological units and delineate some structural elements. Additionally, Principal Component Analysis (PCA) was conducted on Landsat-9 and S1B data, generating PC imagery, which was then used in grey and color modes to highlight lithological contacts with Landsat-9 and to extract linear structures with S1B.

### Fieldwork

**Fig. 3:**
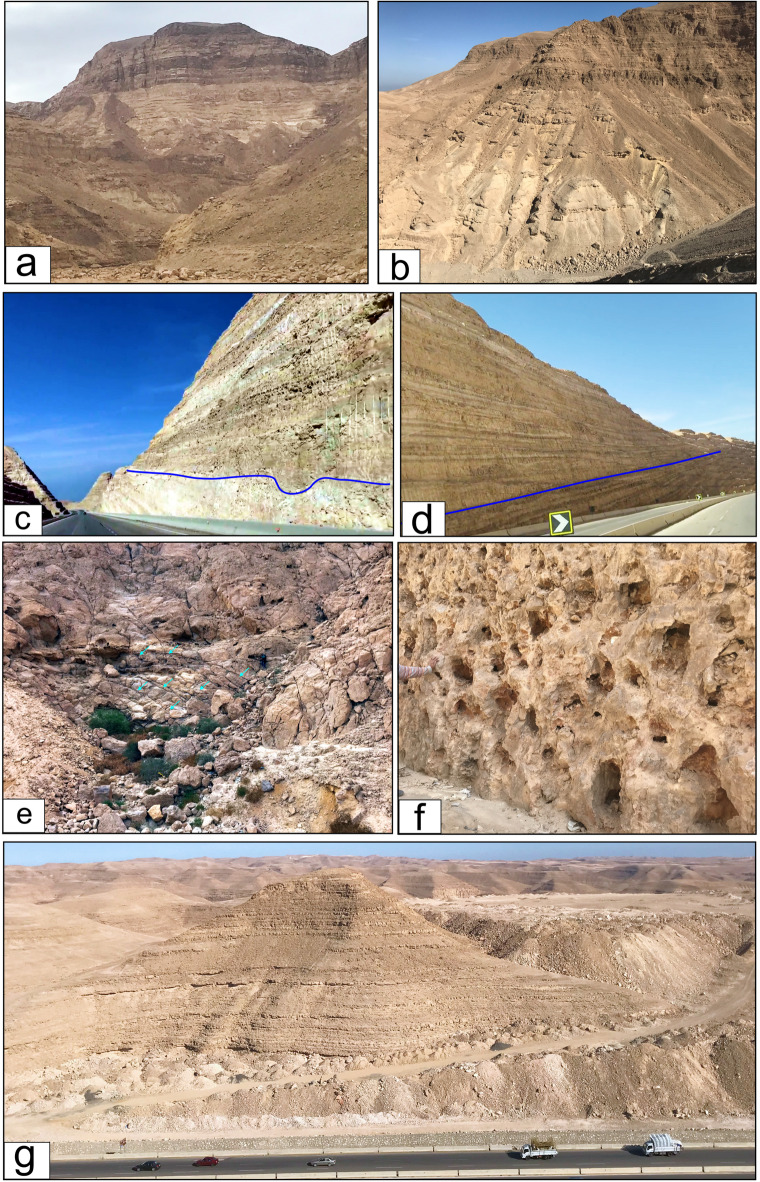
(**a**, **b**) General view of the Northern Galala Formation, (**c**) Contact between the Wadi Al-Rasis and Gebel Ealyan members of the Northern Galala Formation, (**d**) Contact between the Gebel Ealyan and New Galala City members of the Northern Galala Formation, (**e**) Slumped brecciated limestones of the Wadi Al-Rasis member, (**f**) Large vugs and voids with dark grey limestone of the Gebel Ealyan member, (**g**) creamy thin laminated sandy dolostone covered the top of the plateau of the New Galala City Member.

Fieldwork included detailed stratigraphic relationships, sampling representative lithofacies (135 rock samples), and description of bedding features and sedimentary structures from the exposed succession at the top of the Northern Galala Plateau (Fig. 3). Laboratory methods included thin-section petrographic analysis of 52 samples, grain-size analysis of clastic sediments, and identification of fossils. Soil-color chart of^46^ are used to describe the rocks in the field and lab. Facies associations described based on Dunham^47^ classification were interpreted using standard carbonate depositional models^48,49^ and integrated with regional stratigraphic data to establish the depositional framework.

## Results

### Remotely sensed data

#### Lithological discrimination

**Fig. 4:**
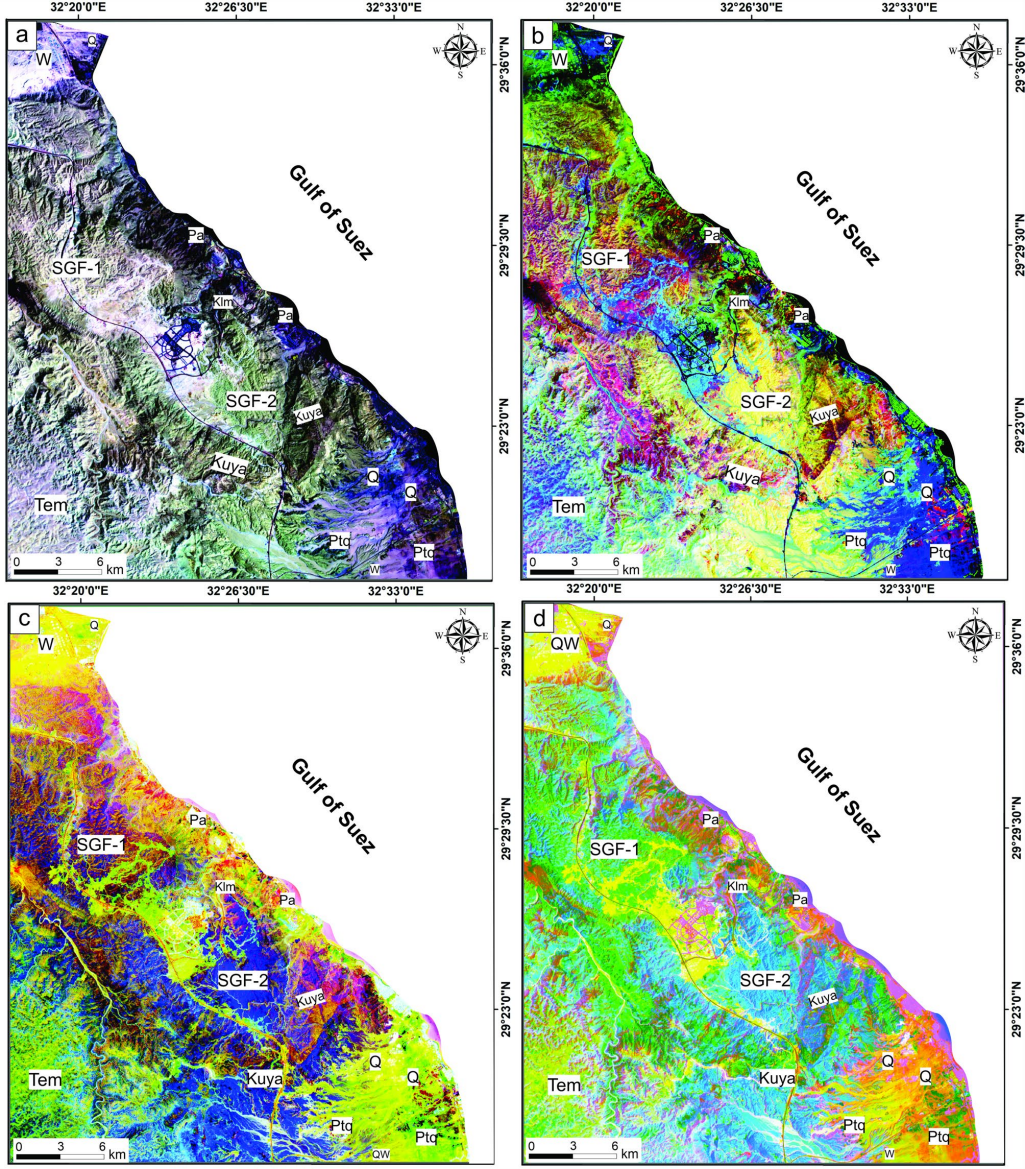
Lithological discrimination utilizing Landsat-9; (**a**) FCC-765, (**b**) BR 6/5 6/7 4/2, (c) PC 234, and d) PC 314 in RGB. These figures were created and processed by ENVI v. 5.6.2. software: https://www.l3harrisgeospatial.com/Software-Technology/ENVI), mainly used for image processing, and ArcGIS Desktop 10.8. (https://www.esri.com/en-us/arcgis/products/arcgis-desktop/overview/).

FCC-765, BR 6/5 6/7 4/2, PC 234 and 314 alongside MNF 234 and 214 of Landsat-9 in RGB mode were selected to discriminate among the lithological units of the study area. Based on the tonal variations, the employed color composited highlighted successfully the sharp and gradual contacts between the sedimentary sequence of the presented area, particularly between the carbonate rocks of the Middle Eocene Mokattam Group (Tem) and Upper Paleocene-Lower Eocene Southern Galala Formation (SGF), which is considered the main target of this research. In the FCC-765 (Fig. 4a), the Mokattam Group is exposed in pale greenish violet, while the carbonate deposits of the SGF exhibit two forms: SGF-1 and SGF-2. The SGF-1 and SGF-2 appear in greenish yellow and pale green to yellowish green, respectively. Moreover, Upper Carboniferous (Pa), Permo-Triassic (Ptq), Upper Cretaceous (Kuya), and Quaternary (Q) rocks, as well as wadi deposits (W), were distinguished by deep violet, greenish blue, deep brown-green, blue, and pale purple colors.

BR 6/5 6/7 4/2-RGB (Fig. 4b) distinguished the Tem, SGF-1, and SGF-2 by blue-cyan yellowish red to brownish green and yellow to yellowish green tone, respectively. The other units, such as Kuya, Ptq, Pa, Lower Cretaceous (Klm), and Q deposits, were discriminated by blood-red to pink, sea-blue, dark black-orange, olive-green, and green-cyan tones, respectively. In both PC 234 and PC 314 **(**Fig. 4c **and d**), the Tem units were featured by olive and yellowish green colors, respectively, which separated them from the SGF-1 and SGF-2, which are distinguished by pinkish orange to deep violet-orange and deep violet to yellowish violet color in PC 234 and appeared in cyan-green and cyan to pinkish cyan in PC 314 respectively. Furthermore, Quaternary and wadi deposits are marked by lemon to yellowish-orange and yellow tones in the PCs mentioned above (Fig. 4c **and d**).

**Fig. 5:**
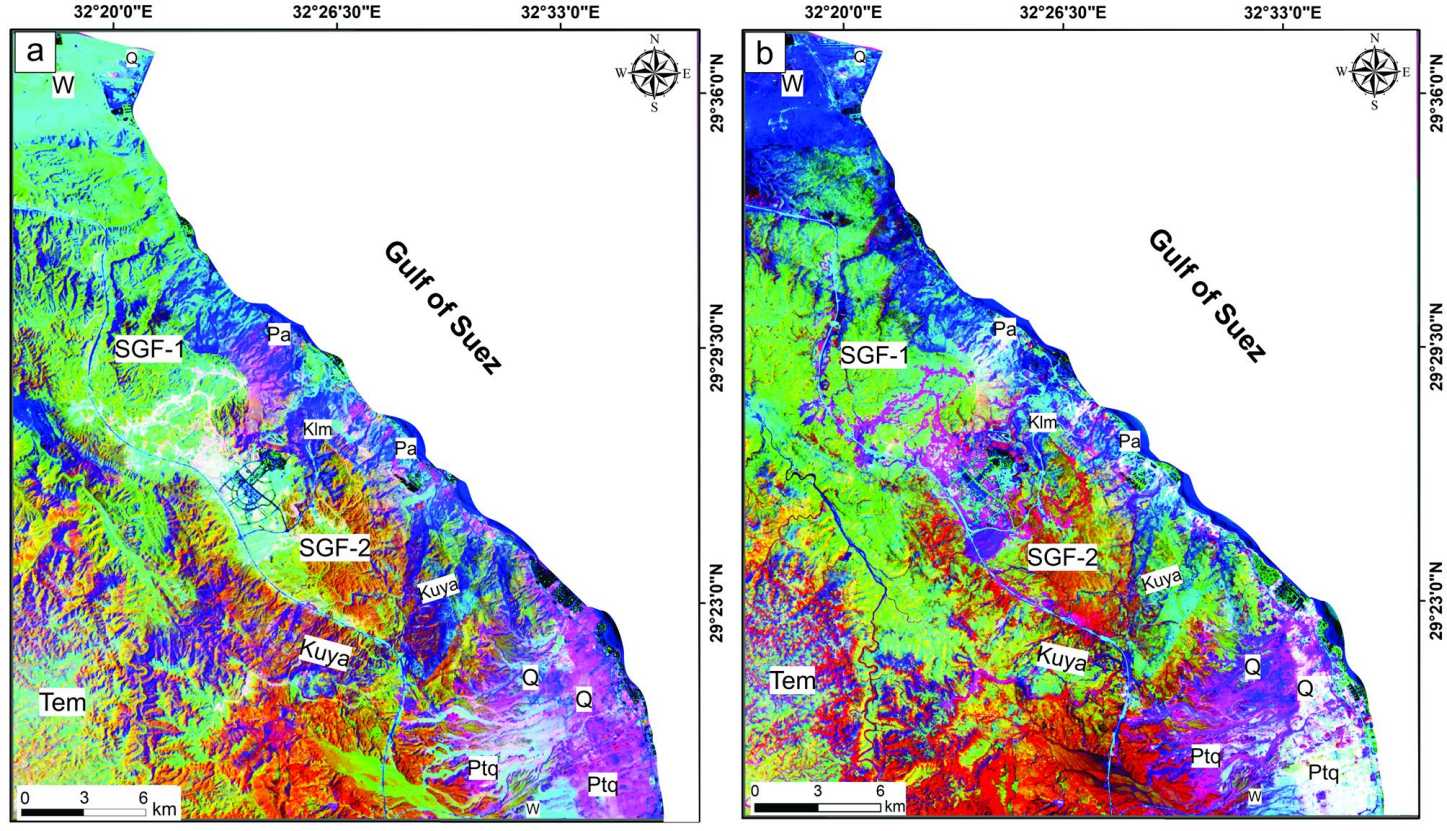
Lithological discrimination utilizing Landsat-9; (**a**) MNF 234 and (**b**) MNF 214 in RGB. These figures were created and processed by ENVI v. 5.6.2. software: https://www.l3harrisgeospatial.com/Software-Technology/ENVI), mainly used for image processing, and ArcGIS Desktop 10.8. (https://www.esri.com/en-us/arcgis/products/arcgis-desktop/overview/).

MNF 234 was able to separate the Tem and SGF units, which were characterized by orange-green for Tem, dark orange for SGF-2, and green-cyan to yellowish green for SGF-1 (Fig. 5a). Also, in MNF 214 (Fig. 5b), bloody red-cyan hues distinguished the Tem unit. At the same time, the SGF-1 appeared yellowish-green, and SGF-2 was distinguished by bloody-red to orange-yellow patches. The remainder of the sedimentary sequence in the presented area was also highlighted with distinct colors in both MNFs (Fig. 5a **and b**).

#### Automatic lineament extraction

**Fig. 6:**
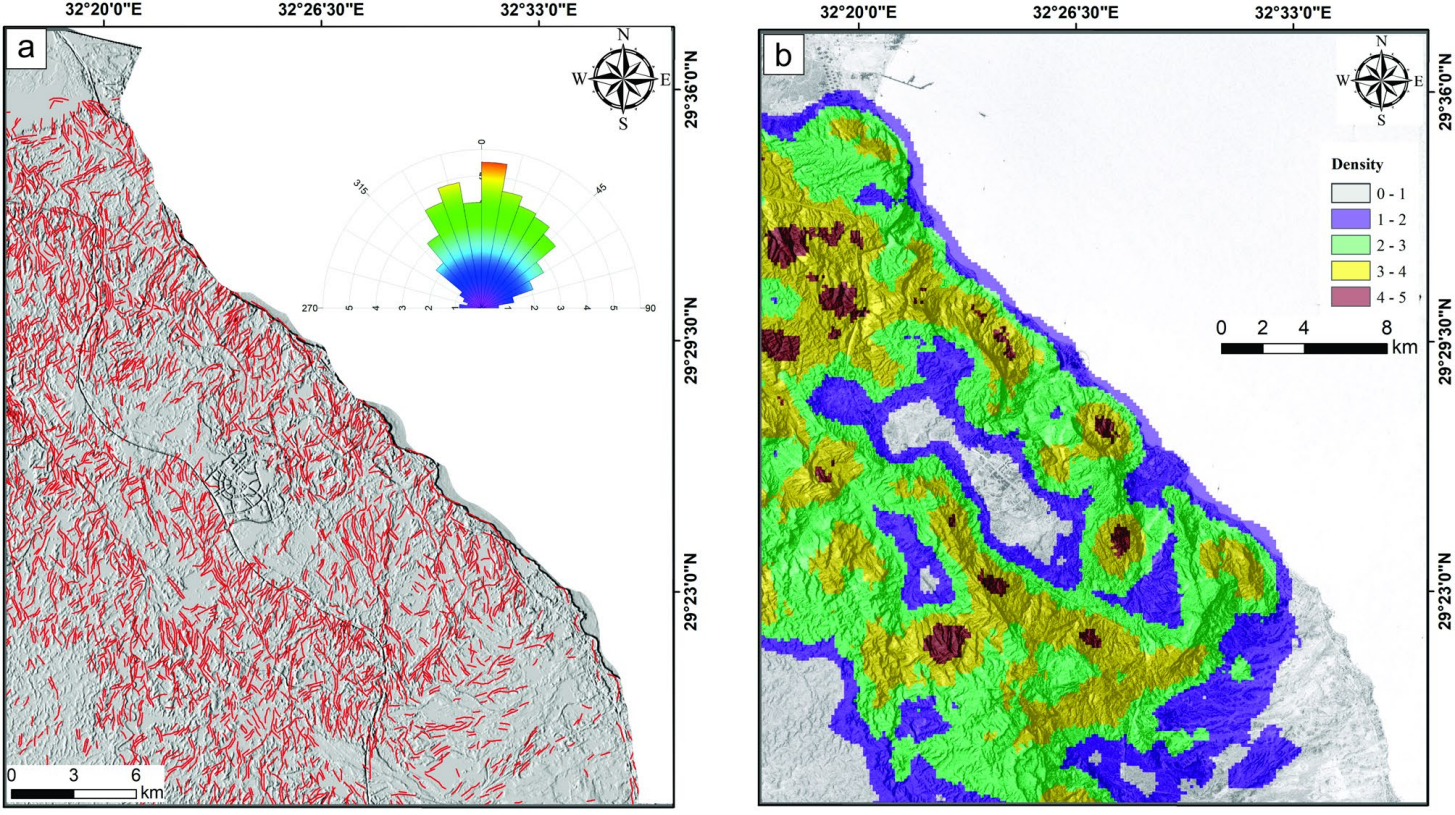
Lineament extraction via radar data of S1B; (**a**) Lineament map and frequency azimuth diagram dropped over Hil shade map, and (**b**) Lineament density display the spatial distribution of lineament concentration per km^2^. These figures were created and processed by ENVI v. 5.6.2. software: https://www.l3harrisgeospatial.com/Software-Technology/ENVI), mainly used for image processing, and ArcGIS Desktop 10.8. (https://www.esri.com/en-us/arcgis/products/arcgis-desktop/overview/).

The identified lineaments (Fig. 6a) were automatically delineated from the grey-scale PC1 of the backscattered Sentinel-1 A (S1B) using PCIgeomatica, resulting in a structural lineament map that illustrates the spatial distribution of linear structures and their domain trends, as shown in an azimuth rose diagram generated with RockWork. The identified lineaments were marked as small, dense red lines scattered across various rock types, indicating a significant lineament density in the northern sector. The azimuth diagram exhibited a range of lineament trends, including NNE, NW, NE and ENE directions, arranged in decreasing order (Fig. 6a). Moreover, the obtained lineaments were processed utilizing the Arc-Map to generate a density map displays the spatial distribution of the lineament concentration over the different sedimentary sequence in the examined area (Fig. 6b). Excluding the urban areas at the center of the study area, the lineament density map revealed that the Mokattam Group (Tem) characterized by low to medium density of lineaments while the Upper Paleocene-Lower Eocene Southern Galala Formation (SGF-1 and SGF-2) are featured by medium to moderately high lineament density (Fig. 6b). However, in general the entire area is characterized by medium to moderately high lineament density.

### Structural analysis

The development of the Galala platform is intricately linked to the activity of the Syrian Arc-Fold-Belt, which formed through the collision of the African and Eurasian Plates. The platform began to emerge during the Campanian-Maastrichtian period, characterized by numerous tectonic uplift events and changes in its morphology, with notable uplift occurring in the Early Eocene^36,39,40,50^. The circum-Tethyan platform stages proposed by Scheibner et al.^51^ primarily emphasize climate-related biotic changes; however, it is essential to recognize that local tectonic factors also play a crucial role in shaping individual platform environments. The development of carbonate platforms under tectonic control is significant across various settings throughout the Phanerozoic^52^. During the Early Eocene, the Egyptian shelf was particularly affected by tectonism, driven by the movement of the African Craton toward Eurasia and the reactivation of Mesozoic fault systems ^36,53,54^. This suggests that the evolution of carbonate platforms in Egypt should be examined from a regional perspective rather than relying solely on the general stages outlined by Scheibner et al.^51^.

The Northern Galala Plateau is characterized by a carbonate ramp that various geological processes have shaped. Carbonate-rich rocks predominantly cover the surface, whereas siliciclastic-rich rocks are along the eastern and southern inclines. Moreover, stratigraphic configuration suggests a repeatedly stacked facies, driven by tectonic movements and sea-level variations, especially during the formation of the Gebel Ealyan member, as shown in the following items. The structural framework of the Northern Galala Plateau is profoundly affected by the tectonic uplift linked to the Syrian Arc Fold Belt. This uplift has resulted in the development of a rimmed platform exhibiting diverse slope angles, with the steep lower slope measuring between 5 and 8 degrees, in contrast to the very gentle ramp slope, which is less than 0.1 degrees^39,55^. The tectonic evolution has produced a complex facies arrangement, indicating sedimentation hiatuses during the Late Cretaceous to Early Paleocene epochs^39,55^. The progression of the carbonate platform is characterized by a transition from a rimmed shelf in the Late Cretaceous to a distally steepened ramp in the latest Cretaceous to Paleocene, ultimately evolving into a homoclinal ramp during the Early Eocene, as exemplified by the carbonate platform of the Northern Galala Plateau. This transition illustrates the impact of changing sea levels and sedimentary dynamics on depositional geometry throughout history^55^.

**Fig. 7:**
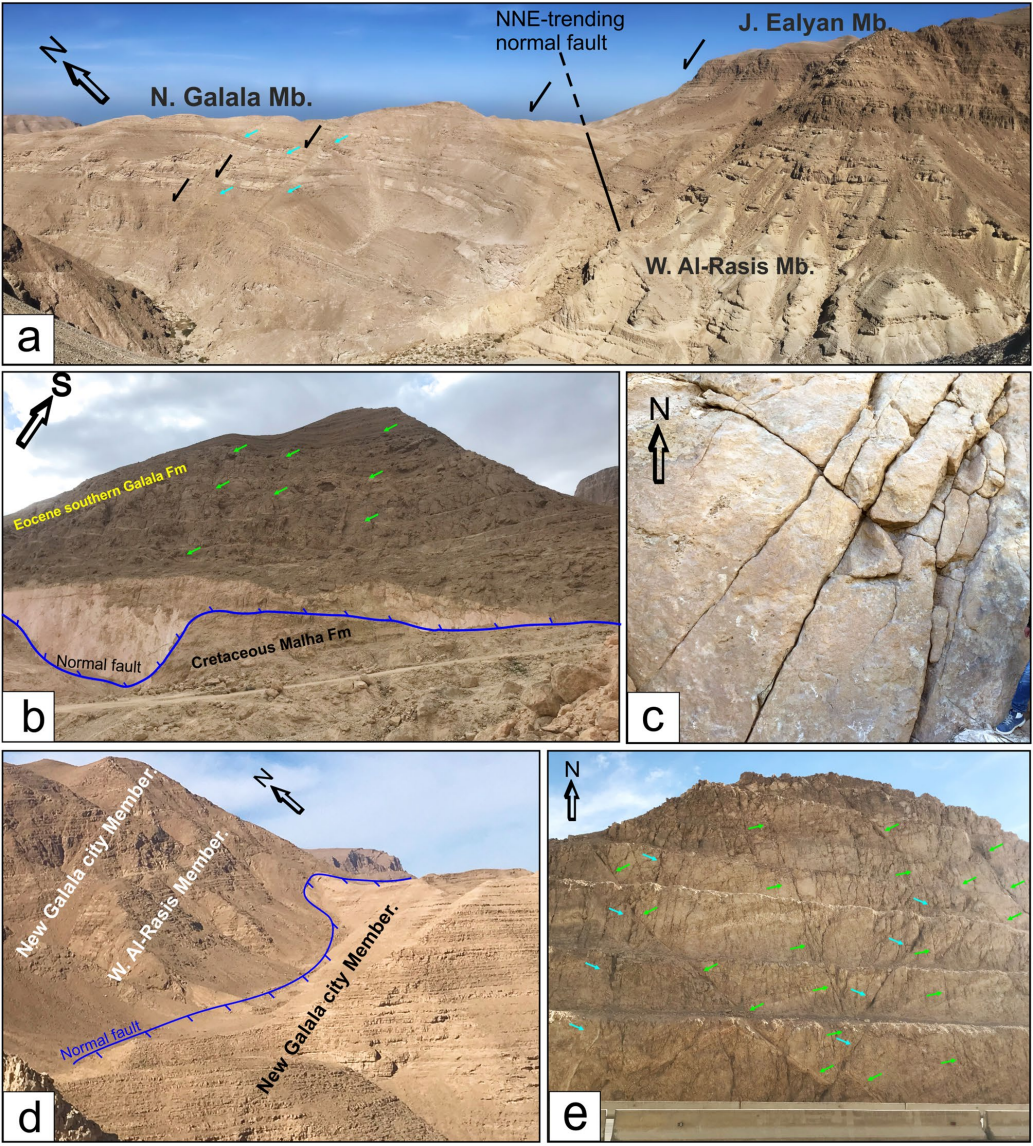
(**a**) NNE-trending step normal faults between the lower and upper member, (**b**) Normal fault between the Eocene southern Galala Formation and Cretaceous Malha Formation, (**c**) NE-trending fractures in the Cretaceous Malha Formation, (**d**) Normal fault between the Gebel Ealyan and New Galala City members of the Southern Galala Formation, (**e**) Conjugate normal faults trending NE-SW and NW-SE in the Southern Galala Formation. The blue arrows indicate faults trending NNE-SSW and NE-SW, while the green arrows denote faults trending NW-SW. The black arrows represent displacement.

The study area is part of the Northern Galala Plateau, located north of Wadi Araba, which is characterized by cliffs on its northern, eastern, and southern sides. The southern cliffs delineate the north boundary of Wadi Araba, while the eastern cliffs offer views of the Gulf of Suez, which lacks a significant coastal plain. The region is predominantly overlain by Eocene carbonates (Fig. 1b) visible in the cliffs, alongside Quaternary fluvial deposits and basaltic rocks^42^. These basaltic rocks were believed to be transported along the primary fault and fractured planes at locations where these planes intersect (Fig. 7a**- d**).

In a regional context, the earlier investigations have concluded that a broad, gently plunging anticline underlies the Galala Plateau and Wadi Araba. In this context, Wadi Araba is situated along the axis of the anticline, while the Northern Galala and Southern Galala Plateaus flank slopes in opposing directions, namly northwest and southeast, respectively^56,57^. Conversely, Wadi Araba is regarded as a significant faulted upthrown block^56^, and its Carboniferous strata have undergone some folding. Furthermore, Höntzsch et al.^36^ deduced that only the northern escarpment of the Southern Galala Plateau exhibits a monocline with its layers inclined southward. At the same time, a fault line delineates the southern escarpment of the Northern Galala, northern of Wadi Araba.

**Fig. 8:**
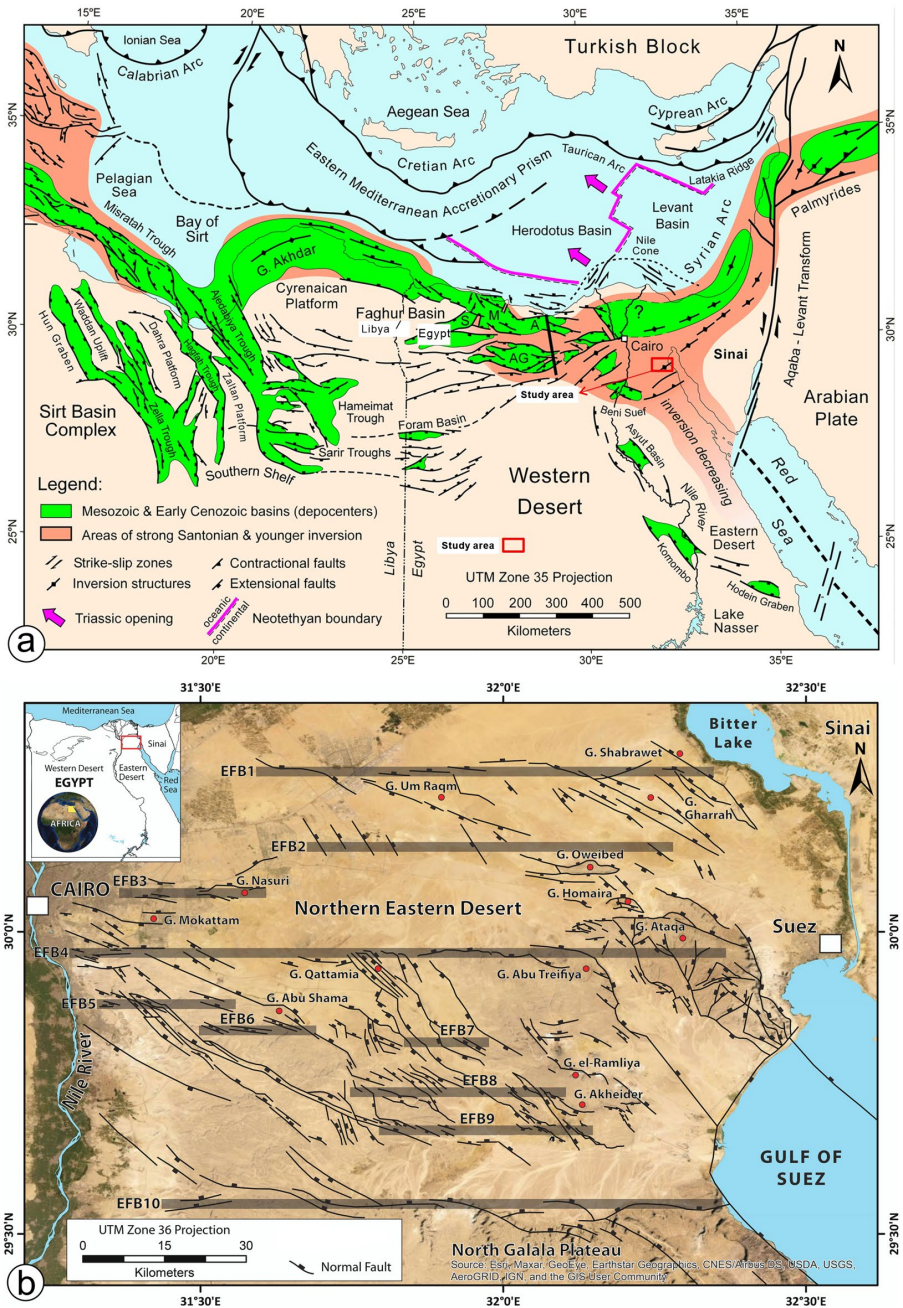
(**a**) Regional geologic setting of northeast Africa and eastern Mediterranean (after Bosworth & Tari^76^. A: Alamein Basin; AG: Abu Gharadig Basin; M: Matruh Basin; S: Shushan Basin. Triassic opening direction and Neotethyan oceanic–continental crustal boundary after Longacre et al.^77^, (**b**) Detailed structural map of the Cairo-Suez District (from Gamal et al., 2021) (after Maqbool et al., 2014, 2016; Moustafa et al., 1985, Moustafa & Abd-Allah, 1992, Moustafa et al., 1998; Henaish, 2018a, 2018b). E-W-elongated left-stepping en echelon fault belts (EFBs) are numbered from north to south and highlighted in gray. UTM—Universal Transverse Mercator. Study area highlighted by yellow lines. Red circles indicate mountains, which translates to Gabal in Arabic, abbreviated as G.

Tectonically, the two Galalas are regarded as a horst while W. Araba is considered a graben due to the NNE-trending normal faults (Fig. 7a), which divide the northern sector of the Eastern Desert^58^. The investigation conducted in the field, along with the linear structures derived from radar data, demonstrated that the study area is influenced mainly by a network of faults and fractures that predominantly trend NNE, NW, and NE (Fig. 7a**- e**), in decreasing order of significance (Fig. 6a). These geological features display cross-cutting interactions and play a crucial role in the evolution of karst formations on the carbonate sediments of the Northern Galala Formation. The Late Cretaceous-Santonian event (84 Ma) continued into the Early Cenozoic time, is considered the most impactful far-field compressional event affecting northern Gondwana to date and responsible for the closure of the Neotethys, which in turn caused a change in regional stress regime (positive structural inversion, Fig. 8a), across the Afro-Arabian plate, from extension to N-S to NW–SE compression with a minor component of strike-slip resulting in the formation of the ‘Syrian Arc’ structures^59–62^. This tectonic event was the main reason responsible for the formation and reactivation of E-W-striking normal faults (Fig. 8b), resulting in the formation of a pop-up structure and fault-propagation ENE-oriented asymmetric anticline fold in the Galala plateaus with a deformed core in W. Araba. The NE-SW inversion fold along W. Araba, south of the study area, is cut through by NW-trending normal faults formed by NE-SW extension that coincided with NW-SE compression related to basin inversion^63,64^. These developed folds and faults have significantly shaped the regional and local characteristics observed in the study area.

Structurally, the generated maps of linear structures (Fig. 6a **and b**) revealed that the Tem group has a lower lineament density than the SGF-1 and SGF-2, which exhibit a moderately higher lineament density. This variation in the surface distribution of liniments among the three units may be related to environmental conditions or to the depth at which each formation was deposited. Typically, the shallow layer near the surface is more susceptible to fracturing than the deeper layer. So, it is supposed that the Tem layers were deposited at a deep depth (deep marine environment). In contrast, the SGF-1 layers were deposited at a shallower depth than the Tem, and SGF-2 was deposited at the shelf area as a transition zone between the two units.

### Lithostratigraphic characters

Lithostratigraphically, the upper Paleocene-lower Eocene carbonate rocks exposed at the top of the Northern Galala Plateau (Figs. 1a, b and c and 2) are classified as Northern Galala Formation^36,37,55,65^. It provides key insights into the paleoenvironmental and tectonic evolution of the southern Tethyan margin during the early Paleogene period. The Southern Galala Formation covers the plateau (Fig. 3a-f), and unconformity overlies the Cretaceous Galala Formation (Figs. 1b and 2**)**, marking a distinct stratigraphic boundary characterized by an erosional or non-depositional hiatus. The present work could differentiate the formation into three formal members, determined through careful field observation of stratigraphic relationships, varying lithofacies, sedimentary structures, depositional aspects, and fossil content. From base to top, these members are the Wadi Al-Rasis Member, the Gebel Ealyan Member, and the New Galala City Member.

**Fig. 9:**
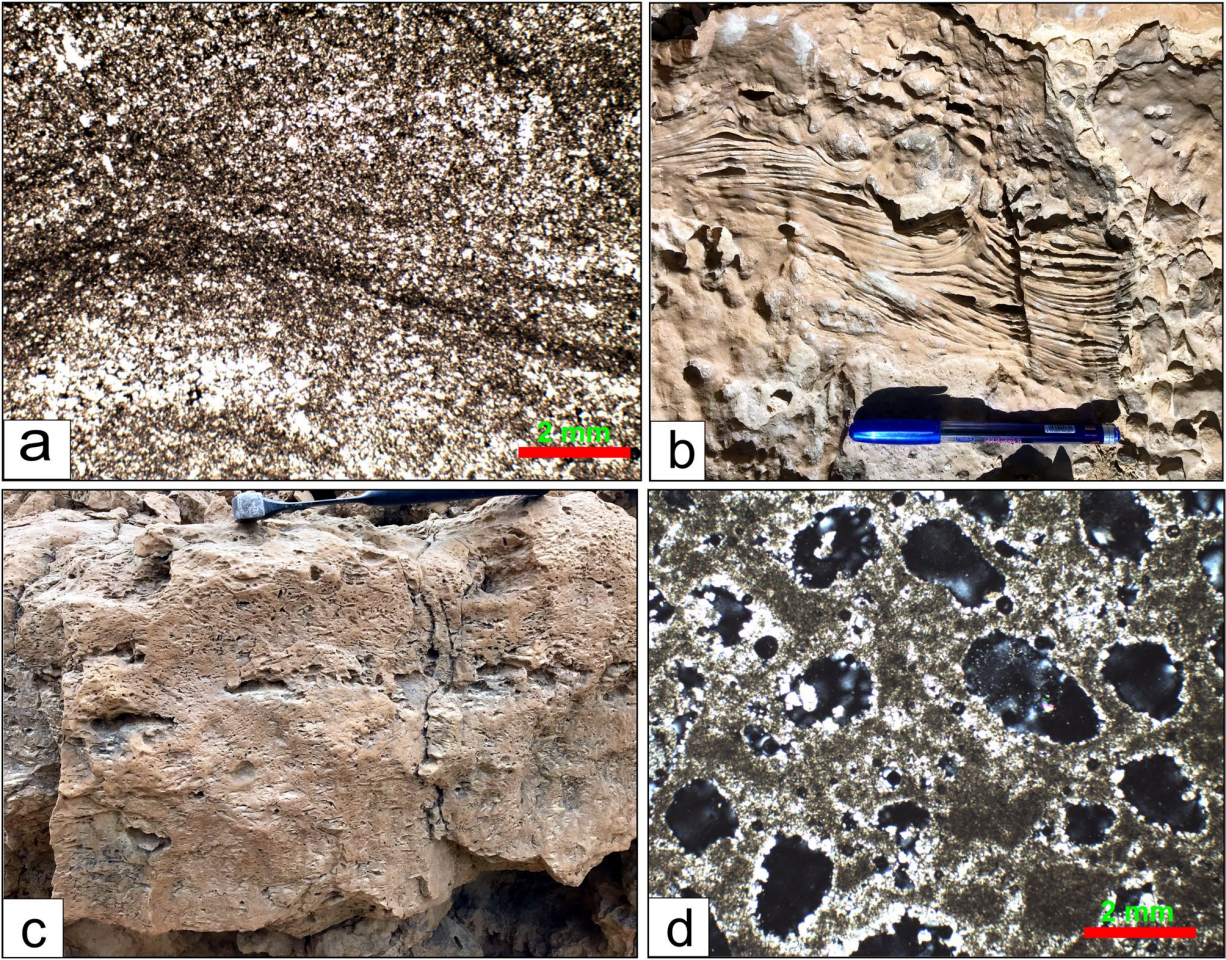
(**a**) Microscopic image shows laminated microbial mudstone microfacies of the photo (**b**). (**b**) Thin laminated (stromatolite) dolostone within the Wadi Al-Rasis Member. (**c**) Fenestrate (bird eye) structure with dolostones of the Gebel Ealyan member. (**d**) The microscopic image shows the fenestrate (bird eye) mudstone microfacies of photo (**c**).

**Wadi Al-Rasis Member**: the “type locality: of this member at the discharge tributaries of the Wadi Al-Rasis (29°32’02.1"N 32°15’31.4” E), north of the Northern Galala Plateau. The thickness of this member shows considerable variation across the area, reaching approximately 70 m at the wadi and decreasing southward. It is composed mainly of very pale brown 10YR (8/2) to light grey (10YR 7/2) thin-bedded dolostone layers (Fig. 3a-c), including varying thicknesses and proportions of nodular limestone, and brecciated carbonate sediments in some parts (Figs. 3e and 9f**)**. However, some distinctive sedimentary structures describe these rocks, e.g., fine, planar to wavy lamination. Another highly diagnostic feature is the presence of a fenestrae structure. These are small, irregular, bubble-like pores or cavities within the sediment. Wadi Al-Rasis Member is equivalent to the lower part of the Northern Galala Formation^36–38,65^. Based on stratigraphic correlation and fossil content (e.g., algae, stromatolites, and ostracods), the Wadi Al-Rasis Member is Late Paleocene in age.

#### Gebel Ealyan member

**Fig. 10:**
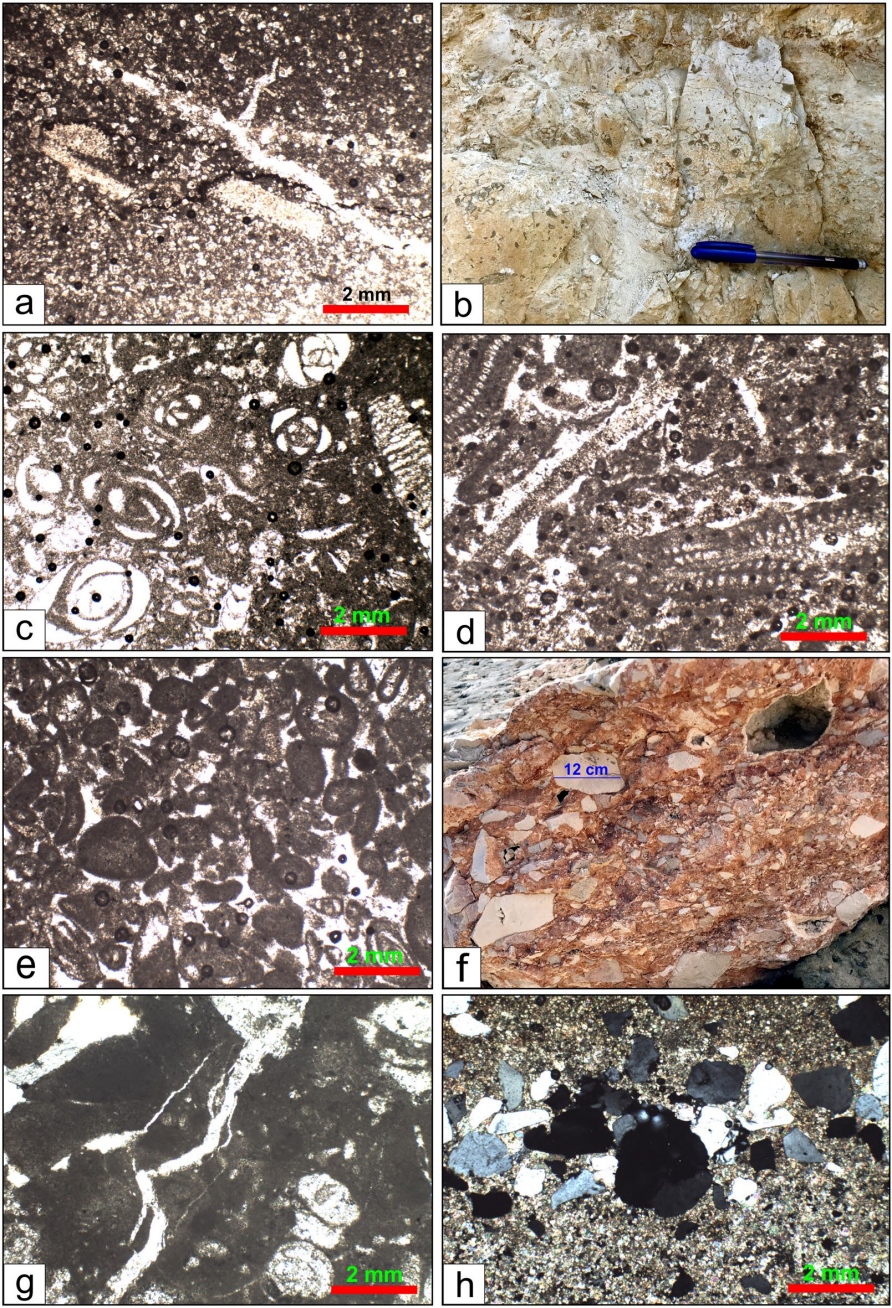
(**a**) Microscopic image shows algal wackestone microfacies that have been subjected to dolomitization diagenesis process, (**b**) Foraminiferal limestone within the Gebel Ealyan member, (**c**) Foraminiferal packstone microfacies of photo (b) rich in *Miliolida* sp., (**d**) The algal wackestone/Packstone microfacies, accompanied by *Alviolina* sp., (**e**) The peloidal packstone/grainstone microfacies. (**f**) brecciated dolostone of the Wadi Al-Rasis Member. (**g**) The intraclasts microfacies of photo (**f**), (**h**) Calcareous quartz arenite microfacies, New Galala City Member.

the “type locality” of this member at the Gebel Ealyan sign, close to the new highway (29°29’55.9"N 32°19’44.5” E). It is 75 m thick, from grey (10Y R6/1) to pale brown (2.5Y 8/2), with thick-bedded limestone units (Figs. 3b-d**)**. These layers are intercalated with basal conglomerates and heterolithic sediments (from ferrigenous mud and gypsum fragments), which cover a well-developed karstification surface (Fig. 3e). The limestone beds display variable textures, from porous to sandy, and host a diverse fossil assemblage, including Early Eocene large benthic Foraminifera, e.g., *Alviolina* sp. *Miliolid* sp., and *Orbitolites* sp. with various types of algae (Fig. 10c-e). Specific horizons exhibit bird’s-eye structures indicative of early diagenetic processes, as shown in Fig. 9c and d. The middle part of the Southern Galala Formation described in the plateau^36,37,65^ is equivalent to the present member.

#### New Galala City member

the New Galala City is constructed within this member at the top of the plateau. It is “type locality” close to the new highway, north-west of the city (29°28’33.9"N 32°19’15.0” E). The upper surface of this member is weathered, showing variability in thickness (ca. 100 m.) across the studied area (Fig. 3d **and g**). It predominantly comprises hard-to-porous pinkish white (10YR 8/2) to very pale brown (10YR 8/2) dolostones. In some parts, thin calcareous sandstone and/or gypsum layers are interbedded with the dolostone rocks. Fossil content of Early Eocene age is sparse, with occasional small nummulites, ostracods, gastropods, and echinoderms. The new Galala City member is equal to the upper part previously studied Northern Galala Formation in the area^36,37,65,66^.

### Microfacies analysis and depositional environments

Microfacies analysis is a crucial tool in the study of carbonate sedimentology. It entails the microscopic investigation of rocks to ascertain their composition, texture, and fossil content. The carbonate platform presents a complex mosaic of sub-environments, each characterized by distinct energy levels, salinity, and water depth. These environmental conditions dictate the types of organisms inhabiting the area and the physical processes responsible for sediment transport and deposition. As a result, the microfacies of these rocks offer an essential record of their depositional history. This work adheres to the widely recognized classification of texture proposed by Dunham^47^ and highlights the grain composition unique to platform environments. The subsequent sections summarize the principal microfacies and their respective interpretations.

#### Laminated microbial mudstone

##### Description

This microfacies is characterized by fine, often wavy, micrite laminations (Fig. 8a). It may contain peloids, and its most diagnostic feature is the presence of fenestral pores (also called bird’s eye pores)—small, irregular voids formed by gas entrapment or the decay of organic material in microbial mats. It is a mud-supported rock (mudstone). However, these facies are recorded from the three studied formation members, especially the Wadi Rasis and New Galala City members.

##### Interpretation

These are the classic microfacies of a tidal flat environment (supratidal and upper intertidal)^49,67,68^. The laminations (Figs. 9a, b**)** represent microbial mats (cyanobacteria), and the fenestral pores indicate frequent subaerial exposure and desiccation^49^.

#### Fenestrate dolostone

##### Description

The fenestrate mudstone is a distinctive carbonate microfacies characterized by a mud-dominated texture containing abundant fenestrae, characterized by small, lens-shaped to irregular pores and voids (Figs. 9c **and d)**. These are typically several millimeters long and are often filled with secondary calcite cement or internal sediment (micrite). It consists of cryptocrystalline or micritic dolomite, suggesting a mud-dominated original sediment. Laminations are usually present and are highlighted by the alignment of fenestrae. The rock is generally massive and lacks precise grading or coarse-grained allochems, such as fossils or intraclasts. Nearly all dolostone layers contain fenestrate structures.

##### Interpretation

The fenestrate dolostone microfacies is a robust indicator of a peritidal environment^49,68^, specifically the upper intertidal to supratidal zones (e.g., a sabkha or saline mudflat) of inner ramp area.

#### Algal Wackestone/Packstone

##### Description

The rock of this microfacies contains a diverse assemblage of skeletal grains floating in a micrite matrix (Fig. 10a). Key grains include fragments of green algae, ostracods, and small gastropods. The texture is typically matrix-supported (wackestone), but in some parts, it is grain-supported with micrite (packstone). Algal facies is observed in the lower part of the Wadi Al-Rasis Member.

##### Interpretation

The high micrite content in wackestone texture are clear indicators of a low-energy setting, below the influence of regular wave action^48^. According to Flugel^49^ the presence of the algae and ostracods within this microfacies is interpreted to have been deposited in a low-energy, protected subtidal to shallow lagoonal environment of the inner ramp region.

#### Foraminiferal Packstone/Wackestone

**Description**: These microfacies have low diversity and are dominated by a specific group of Foraminifera: *Miliolida* and/or other perforate forams, e.g., *Alviolina* (Fig. 10b, c, **and d**). These grains are often associated with peloids and intraclasts. The matrix is micrite with sparrite in parts, and the texture ranges from wackestone to packstone. The limestones of the Gebel Ealyan Member host these facies with different foraminiferal taxa.

##### Interpretation

Miliolid foraminifera are tolerant of elevated salinity and restricted conditions. Therefore, these microfacies indicate a restricted lagoon^68^ with limited water circulation, higher-than-normal salinity, and generally quiet water conditions^48,49^.

#### Peloidal Packstone/Grainstone

##### Description

This microfacies is dominated by peloids (micritized fecal pellets or micritized grains) with minor skeletal debris (Fig. 10e). The texture is grain-supported, and the interstitial space is filled with either micrite (packstone) or sparry calcite cement (grainstone). The peloids are typically well-sorted and sub-rounded. Peloidal Packstone/Grainstone microfacies is observed only in the Gebel Ealyan Member.

##### Interpretation

Peloids are often associated with bioturbation and reworking. These microfacies form in moderate-energy environments, such as lagoon margins or protected shoals^48^ where currents are strong enough to winnow away micrite but insufficient to transport larger skeletal fragments^49^.

#### Intraclastic Rudstone/Floatstone

##### Description

These microfacies are dominated by angular to rounded fragments of reworked, lithified, or semi-lithified carbonate sediment (Figs. 10f **and g**). These intraclasts are typically micritic, indicating they were ripped from a low-energy environment like a tidal flat or lagoon floor. The intraclasts are supported by a matrix of finer grains and micrite (floatstone) or are clast-supported (rudstone). A thick succession of this facies is noted at the fault contact of the Southern Galala Formation with Cretaceous rocks.

##### Interpretation

Intraclasts form through erosion of previously deposited sediment. These microfacies are common in tidal channels or as storm deposits (tempestites), where strong, episodic currents erode the seafloor and redeposited the fragments in the same area^48^ which so-called basal conglomerates.

#### Calcareous quartz arenite

##### Description

Calcareous quartz arenite is a variety of quartz arenite sandstone, and the primary non-detrital cement is calcite. Framework grains constitute about 90% of the rock volume by ultra-stable mono- and poly-crystalline quartz grains (Fig. 10h). The grains are well-rounded and well-sorted, indicating extensive weathering, reworking, and high textural maturity. The interstitial pore space between the quartz grains is filled primarily by sparry calcite and/or dolomitic cement. This microfacies constitutes the siliciclastic sediments within the dolostones of the New Galala City Member.

##### Interpretation

The microfacies component’s high textural and compositional maturity points to prolonged transport (e.g., eolian or beach environments) or repeated reworking in a high-energy setting, such as a shallow marine shelf or a tidal sand bar^69^.

### Diagenetic processes and economic aspects

The carbonate skeletons of the ramp are predominantly made up of aragonite and high-Mg calcite, both of which exhibit high reactivity and are prone to diagenetic changes. The diagenetic process initiates within the marine phreatic environment. It is often affected by meteoric waters, owing to the shallow nature of the depositional environment and the subsequent fluctuations in sea level^49^. The diagenetic evolution of inner ramp carbonates encompasses a complex series of frequent competing processes as outlined below:

#### Micritization

In the micritization process, the margins of the carbonate skeletons are replaced by microcrystalline carbonate crystals called “micrite”. The process involves microbes attacking the outside of grains by boring small holes. Later, these holes are filled with micrite cement to form a micrite envelope^70^. Most of the original fossil foraminifer and calcareous algal skeletons in Fig. 9c and d are micritized and filled with sparry calcite.

#### Cementation

This phenomenon occurs swiftly on the seafloor or beneath it and is distinguished by isopachous fringes composed of fibrous aragonite or high-Mg calcite. This process quickly eliminates primary porosity, forming hardgrounds and diminishing reservoir potential^71^. Conversely, aragonite and high-Mg calcite exhibit thermodynamic instability in fresh, meteoric water. These minerals easily dissolve when exposed (for instance, during periods of low sea level), increasing the primary porosity (Fig. 10f).

#### Dolomitization

The settings of inner ramps serve as key sites for dolomitization models, owing to their shallow characteristics and interaction with evaporative fluids. In supratidal areas, the process of evaporative pumping facilitates the movement of Mg-rich brines through the sediment, leading to the replacement of CaCO₃ with dolomite^71^. This process generally results in the formation of fine-crystalline dolomite that retains its fabric (Fig. 10a). Dolomitization has the potential to generate considerable secondary intercrystalline porosity through a net volume reduction of up to 13%^49^, while also fostering a more stable mineralogy that is resistant to compaction.

#### Compaction

Compaction typically manifests as mechanical or chemical types^71^. In mechanical compaction, grain reorientation and breakage happen early, decreasing porosity (Fig. 10e). Conversely, chemical compaction, also known as pressure dissolution, initiates at considerable depths where stress induces dissolution at grain contacts, forming stylolites and along seams^49^.

#### Recrystallization and neomorphism

Refer to the in-situ alteration of a mineral into a more stable variant. A prevalent example of this process is the transformation of aragonite into calcite^49^, which often occurs as a pseudomorphic replacement that preserves the original structure (Fig. 8d).

Economically, some of the diagenesis processes significantly modifies the rock strength. Compaction, cementation and neomorphism usually enhance rock strength and brittleness. Furthermore, dolomitization produces a more brittle rock and results in a multi-colored appearance, unlike limestone, which is more ductile. These processes reinforce the strength and geomorphological shape of the carbonate rocks to be suitable for construction, decoration, and developing new highways of the New Galala City. On the other hand, pressure dissolution, recrystallization, dolomitization, and stylolitization generate planes of weakness and/or voids that can influence the porosity, fracture propagation, and compartmentalization within the rock mass. The newly formed rock products often construct hydrocarbon reservoirs and/or underground water aquifers in the studied region.

## Discussion

### Geological and structural characteristics of the inner carbonate platform

The use of Landsat-9 image color variations successfully distinguished sedimentary layers and improved the geological map by highlighting contacts between formations. A key challenge was differentiating rock units covered by sediments with similar reflectance. Notably, the study differentiated the Middle Eocene Mokattam Group (Tem) from the Upper Paleocene-Lower Eocene Southern Galala Formation (SGF), which are often mapped as a single unit. Processed images and fieldwork revealed unique colors and facies for each. The SGF was further split into SGF-1 and SGF-2.

The evolution of the Galala platform is closely connected to the Syrian Arc-Fold-Belt, which formed from the collision of the African and Eurasian tectonic plates and experienced significant uplift during the Campanian-Maastrichtian and Early Eocene epochs. Although regional platform stage models highlight biotic changes, local tectonic factors such as the movement of the African craton and the reactivation of Mesozoic fault systems have been crucial in shaping Egypt’s carbonate platforms^72,73,74^.

The Northern Galala Plateau mainly consists of Eocene carbonates affected by faulting and fracturing. Geological interpretations reveal the presence of anticlines, horsts, and grabens related to a series of NNE faults. A major compressional event during the Late Cretaceous (Santonian) period is a key factor in forming the Syrian Arc structures, leading to subsequent folding and faulting in the area. Additionally, variations in lineament density across different geological units suggest distinct depositional environments and various levels of fracturing exposure (Fig. 6).

### Lithostratigraphy and environmental evolution of the studied carbonate rocks

The lithostratigraphic classification of lower Paleogene carbonate sediments in the Northern Galala Plateau remains contentious. Stratigraphers are divided between assigning these units to the established Thebes and Mokattam groups^35,44^ and classifying them as a specific unit, the Southern Galala Formation^36,41,42,75^. Integrated evidence from field observations, stratigraphic investigations, fossil assemblages, structural characteristics, and remote sensing data favors the interpretation of these sediments as the Southern Galala Formation, rather than as part of the Thebes and Mokattam groups. Moreover, the present work introduced three formal members (Wadi Al-Rasis, Gebel Ealyan, and New Galala City) for the Southern Galala Formation, replacing the previous informal members.

The color differences in Landsat-9 images between Tem, SGF-1, and SGF-2 are related to depositional carbonate ramp position: Tem was deposited in the outer-, SGF-1 in the inner-, and SGF-2 in the mid-ramp environments.

Eustatic sea-level fluctuations and the activity of nearby tectonic regions heavily influence the development and evolution of carbonate platform systems^36^. Based on Paleocene data from the Galala platform, Scheibner et al. ^39^ propose that local tectonic processes have a greater effect on the platform’s development than sea-level changes driven by eustatic factors. The effects of these processes are preserved within the Southern Galala Formation, particularly in the Gebel Ealyan Member, as repeated, cyclical facies stacking. This member exhibits a progradational microfacies sequence, beginning with wackestone, packstone, and grainstone (Figs. 2 and 10). These facies were subsequently and rapidly exposed to subaerial unconformity and karstification due to tectonic uplift. Consequently, this tectonic activity explains why the lineament structures developed in the Gebel Ealyan Member are more extensive than in other parts of the studied area (Fig. 6).

Scheibner et al.^39^ identified five stages of platform development related to the progradation and retrogradation of the platform margin, illustrating the evolution of the study area from Maastrichtian to earliest Eocene (stages A–E). A subsequent phase (stage D) saw debris flows fill the accommodation space at the platform edge, leading to a southward migration of the platform margin. During this period, the Wadi Al-Rasis Member was deposited unconformably over Cretaceous rocks in the studied area. Stage E reflects platform retrogradation during the latest Paleocene, just before the Paleocene-Eocene Thermal Maximum (PETM), suggesting sea-level rise. This retrogradation involved extensive carbonate deposition and the development of Large Benthonic Foraminifera (LBF) shoals within Gebel Ealyan and New Galala City members. Additionally, pronounced sea-level fluctuations and intense tectonic activity during these stages likely prevented the formation of evaporite deposits within the succession.

## Conclusions

The Lower Paleogene The inner carbonate ramp of the Southern Galala Formation (SGF) in the Northern Galal Plateau was investigated for the first time by remote sensing techniques, besides the stratigraphic and structural aspects.

- Remote sensing techniques effectively distinguished carbonate layers and separated the often-combined Mokattam Group and Southern Galala Formation into distinct units based on their unique spectral and facies characteristics. The Southern Galala Formation was further subdivided into two subunits (SGF-1 and SGF-2). Landsat-9 color differences map the paleogeography of a carbonate ramp, distinguishing the outer-ramp (Tem), inner-ramp (SGF-1), and transitional mid-ramp (SGF-2) environments.
- The Northern Galala Plateau structure is characterized by anticlines, horsts, and grabens formed by NNE-trending faults, primarily resulting from Late Cretaceous (Santonian) compression that created the Syrian Arc. Furthermore, differences in lineament density among the rock units indicate varying depositional settings and degrees of fracturing. Moreover,
- Lithostratigraphically, the Southern Galala Formation is divided into three members, each with distinct lithology and depositional settings. The Wadi Al-Rasis Member consists of thin, pale dolostones with thin laminated microbial mudstones. The Gebel Ealyan Member is made of cyclic, fossil-rich grey limestones that underwent karstification. The New Galala City Member features creamy fossiliferous dolostone with siliciclastic layers.
- Analysis of fossils, rock types, and microfacies indicates the upper and lower members were deposited in tidal flats, while the middle member (Gebel Ealyan) formed in a restricted lagoon to shoal environment on an inner ramp. These inner-ramp deposits were heavily altered by diagenetic processes such as cementation and dolomitization.
- Diverse fossil content within the inner ramp deposits, primarily characterized by larger benthic foraminifera (LBF) alongside other benthic organisms such as stromatolites, green algae, coralline red algae, and ostracods. The existence of these species suggests warm, shallow-marine conditions in low latitudes. The fossil assemblages play a vital role in comprehending the paleoenvironmental conditions, such as Paleocene-Eocene Thermal Maximum (PETM), and the biological diversity that prevailed during the Eocene epoch.
- The evolution of carbonate platforms in the studied area is influenced by both eustatic sea-level changes and regional tectonics, with evidence suggesting tectonics were the dominant control. This is recorded in the Gebel Ealyan Member, which shows repeated, prograding facies and paleosol cycles that were abruptly exposed and karstified due to tectonic uplift. This intense tectonic history also explains why the Gebel Ealyan area has more extensive fault and fracture (lineament) networks than other areas.

## Data Availability

The datasets used and/or analysed during the current study are available from the corresponding author on reasonable request.

## References

[1] Read, J. F. Carbonate platform facies models. *AAPG Bulletin* 69, 1–21. 10.1306/AD461B79-16F7-11D7-8645000102C1865D (1985).

[2] Tucker, M. E. & Wright, V. P. Carbonate Sedimentology. Blackwell Scientific Publications, Oxford, 482pp. DOI:10.1002/9781444314175 (1990).

[3] Papakonstantinou, M., Sergiou, S., Geraga, M., Prandekou, A., Dimas, X., Fakiris, E., Christodoulou, D., Papatheodorou, G. Sedimentological, Geochemical, and environmental assessment in an Eastern Mediterranean, stressed coastal setting: the Gialova Lagoon, SW Peloponnese, Greece. *Water* 16, 2312. 10.3390/w16162312 (2024).

[4] Marshall, J.C., Tibby, J., Moss, P., Martin, H., Gontz, A., Lau, A., Jacobsen, G.E., Cadd, H., Gadd, P.S., Negus, P., Mcgregor, G.B., Hofmann, H., Schulz, C., Barr, C., Maizma, S., Hotchkis, M., Cloutier, N. High-resolution analysis of sediments from eighteen Mile swamp (eastern Australia) records its transition from a fluctuating coastal lagoon to stable freshwater swamp. *Journal of Quaternary Science* 40, 684–710. 10.1002/jqs.3677 (2024).

[5] Flügel, E. Microfacies of Carbonate Rocks: Analysis, Interpretation and Application. Springer-Verlag, Berlin Heidelberg, 976pp (2004).

[6] James, N.P. Shallowing-Upward Sequences in Carbonates. In: Walker, R.G. (Ed.), Facies Models (2nd ed.). Geoscience Canada Reprint Series 1, *Geological Association of Canada* 213–228 (1984).

[7] Perović, M., Obradović, V., Zuber-Radenković, V., Mitrinović, D., Knoeller, K. & Turk Sekulić, M. Integrated analysis of ammonium origins in a Serbian anoxic alluvial aquifer: insight from physicochemical, isotopic, Microbiological data. *Applied Geochemistry* 171, 106103. 10.1016/j.apgeochem.2024.106103 (2024).

[8] Farouk, S., Fagelnour, M., Zaky, A.S., Arafat, M., Salama, A., Al-Kahtany, K., Gentzis, T., Jovane, L. Petroleum System Evaluation: Hydrocarbon Potential and Basin Dynamics in Abu Darag Sub-Basin, Northern Gulf of Suez (Egypt). *Minerals* 14, 1154. 10.3390/min14111154 (2024).

[9] Nait-Hammou, H., El Khalidi, K., Makaoui, A., Chierici, M., Jamal, C., Mejjad, N., Khalfaoui, O., Salhi, F., Idrissi, M. & Zourarah, B. Environmental factors driving carbonate distribution in marine sediments in the Canary current upwelling system. *J. Marine Science and Engineering* 13, 1709. 10.3390/jmse13091709 (2025).

[10] Zhang, J., Hu, J., Liu, B. & Zhang, X. The genesis mechanisms, geological characteristics, and exploration and development of deep-sea carbonate reservoirs: current status and future prospects., *Advances in Resources Research*. 10.50908/arr.5.3_1133 (2025).41292700

[11] Kakemem, U., Jafarian, A., Husinec, A., Adabi, M.H. & Mahmoudi, A. Facies, sequence framework, and reservoir quality along a triassic carbonate ramp: Kangan Formation, South Pars Field, Persian Gulf Superbasin. *Journal of Petroleum Science and Engineering* 198, 108166. 10.1016/j.petrol.2020.108166 (2021).

[12] Zhu, Y., Zheng, J., Zhang, J., Luo, X., Yu, G., Li, J., Hu, F., & Yang, G. Facies, Depositional environment and reservoir quality of an early cambrian carbonate ramp in the Tarim Basin, NW China. *Minerals* 13, 791. 10.3390/min13060791 (2023).

[13] Al-Qayim, B.A. The paleogene forebulge carbonate Banks, Zagros foreland basin, Northern iraq: their Paleogeography, basin Evolution, and economic implications. *The Iraqi Geological Journal* 230–253. 10.46717/igj.57.2c.16ms-2024-9-24 (2024).

[14] Veysi, H., Taghabi, S.J., Babaiy, M., Tafazoli, M. Lead and barium strata-bound deposits in eocene carbonate ramps of iran: implications for the influence of sedimentary environment characteristics on the distribution of ore reserves. *Ore and Energy Resource Geology* 18, 100073. 10.1016/j.oreoa.2024.100073 (2025).

[15] Cantrell, D.L., Swart, P.K., Handford, C.R., Kendall, C., & Westphall, H. Geology and production significance of dolomite, Arab-D reservoir, Ghawar field, Saudi Arabia. *GeoArabia*, p. 45–60.der Universität Bremen, v. 112, p. 1–135 (2001).

[16] El-Yamani, M.S., John, C.M., Bell, R. Stratigraphic evolution and karstification of a cretaceous Mid‐Pacific Atoll (Resolution Guyot) resolved from core‐log‐seismic integration and comparison with modern and ancient analogues. *Basin Research* 34, 1536–1566. 10.1111/bre.12670 (2022).

[17] Zerga, B. Karst topography: Formation, processes, characteristics, landforms, degradation and restoration: A systematic review. *Watershed Ecology and the Environment* 6, 252–269. 10.1016/j.wsee.2024.10.003 (2024).

[18] Al-Hashim, M.H., Al-Aidaros, A. & Zaidi, F.K. Geological and Hydrochemical Processes Driving Karst Development in Southeastern Riyadh, Central Saudi Arabia. *Water* 16, 1937. 10.3390/w16141937 (2024).

[19] Pour, A.B., Ranjbar, H., Sekandari, M., Abd El-Wahed, M.A., Hossain, M.S., Hashim, M., Yousefi, M., Zoheir, B., Wambo, J.D.T. & Muslim, A.M. Remote sensing for mineral exploration., Geospatial Analysis Applied to Mineral Exploration. 10.1016/b978-0-323-95608-6.00002-0 (2023).

[20] Aboelkhair, H., Abdelhalim, A., Hamimi, Z. & Al-Gabali, M. Reliability of using ASTER data in lithologic mapping and alteration mineral detection of the basement complex of West Berenice, southeastern Desert, Egypt. *Arabian J. Geosci.* 13, 287 (2020).

[21] Shebl, A., Badawi, M., Dawoud, M., Abd El-Wahed, M., El-Dokouny, H.A. & Csámer, Á. Novel comprehensions of lithological and structural features gleaned via Sentinel 2 texture analysis. *Ore Geology Reviews* 168, 106068. 10.1016/j.oregeorev.2024.106068 (2024).

[22] Kamel, M., Abdeen, M.M., Youssef, M.M., Orabi, A.M. & Abdelbaky, E. Utilization of landsat-8 (OLI) image data for geological mapping of the neoproterozoic basement rocks in the central Eastern desert of Egypt. *J. Indian Soc. Remote Sens.* 50 (3), 469–492 (2022).

[23] Abd El-Wahed, M.A. & Thabet, I.A., 2017. Strain geometry, microstructure and metamorphism in the dextral transpressional Mubarak Shear Belt, Central Eastern Desert, Egypt. *Geotectonics* 51(4), 438–462 (2017).

[24] Abd El-Wahed, M.A., Lebda, E., Ali, A., Kamh, S. & Attia, M. The structural geometry and metamorphic evolution of the Umm Gheig shear belt, central Eastern Desert, egypt: implications for exhumation of Sibai core complex during oblique transpression. *Arab J Geosci* 12, 764. 10.1007/s12517-019-4760-y (2019).

[25] Abd El-Wahed, M., Kamh, S., Abu Anbar, M., Zoheir, B., Hamdy, M., Abdeldayem, A., Lebda, E.M. & Attia, M. Multisensor Satellite Data and Field Studies for Unravelling the Structural Evolution and Gold Metallogeny of the Gerf Ophiolitic Nappe, Eastern Desert, Egypt. *Remote Sensing* 15, 1974. 10.3390/rs15081974 (2023).

[26] Abd El-Wahed, M.A., Eldosouky, A.M., Kassem, O.M.K., Abo-Rayan, A.A. & Attia, M. Kilometer-scale hook-shaped Type2/Type3 folds due to refolding and transpressional strike-slip reversal in the Egyptian Nubian Shield, East African orogenic belt. *Precambrian Research* 429, 107912. 10.1016/j.precamres.2025.107912 (2025a).

[27] Abd El-Wahed, M.A., Kamh, S., Attia, M. & Eldosouky, A.M. Structural geometry and gold ores along the first outlined N–S dextral shear zone in the Egyptian Nubian Shield, East African orogenic belt: New insights from integrated remote sensing, gravity, magnetic and field studies. *Geomechanics and Geophysics for Geo-Energy and Geo-Resources* 11, 76. 10.1007/s40948-025-01003-8 (2025).

[28] Eldosouky, A.M., Abd El–Wahed, M.A., Saada, S.A. & Attia, M. New Insights into Structural and Tectonic Evolution of Safaga-Semna Shear Belt: Advanced Integration of Aeromagnetic, Remote Sensing and Field Studies. *Geomech Geophys Geo-energ Geo-resour* 11, 31. 10.1007/s40948-025-00946-2 (2025).

[29] Eldosouky, A.M., El-Wahed, M.A.A., Attia, M., Saada, S.A., & Abbas, M.A. Advanced integrated strategy for structural and mineralogical exploration of inaccessible regions employing remote sensing and multiscale analysis of aeromagnetic data. *Scientific Reports* 15, 31205. 10.1038/s41598-025-16618-w (2025). 40855173 10.1038/s41598-025-16618-w PMC12379228

[30] Zoheir, B., Abd El-Wahed, M.A., Pour, A.B. & Abdelnasser, A. Orogenic gold in transpression and transtension zones: field and remote sensing studies of the Barramiya–Mueilha sector, *Egypt. Remote Sensing* 11, 2122. 10.3390/rs11182122 (2019).

[31] Zoheir, B., Emam, A., Abd El-Wahed, M. & Soliman, N. Gold endowment in the evolution of the Allaqi-Heiani suture, egypt: A synthesis of geological, structural, and space-borne imagery data. *Ore Geology Reviews* 110, 102938. 10.1016/j.oregeorev.2019.102938 (2019).

[32] Abd El-Wahed, M.A., Kamh, S.Z. Evolution of Strike-Slip duplexes and Wrench-Related folding in the central part of al Jabal al Akhdar, NE Libya. *The Journal of Geology* 121, 173–195. 10.1086/669249 (2013).

[33] Abd El-Wahed, M.A. Shear-related gold mineralization in the Egyptian Nubian Shield, East African orogenic belt. *Mediterranean Geoscience Reviews* 7, 185–253. 10.1007/s42990-025-00168-4 (2025).

[34] Abd El-Wahed, M., Attia, M. Transpressional inversion and fold superimposition in the Southern Eastern desert of Egypt, East African orogenic belt. *Journal of the Geological Society* 182. 10.1144/jgs2024-235 (2025).

[35] Abdelazeem M, Fathy, M. S. & Khalifa, M. M. Integrating magnetic and stratigraphic data to delineate the subsurface features in and around new Galala City, Northern Galala Plateau, Egypt. NRIAG *Journal of Astronomy and Geophysics* 8 (1), 131–143 (2019).

[36] Höntzsch, S., Scheibner, C., Kuss, J., Marzouk, A.M. & Rasser, M.W. Tectonically driven carbonate ramp evolution at the Southern Tethyan shelf: the lower eocene succession of the Galala mountains, Egypt. *Facies* 57, 51–72. 10.1007/s10347-010-0229-x (2010).

[37] Abd-Elhameed, S., Mahmoud, A.A. & Salama, Y. Late Paleocene–Early eocene larger foraminifera from the Galala Plateaus, North Eastern Desert, egypt: biostratigraphic, paleoenvironmental and paleoecological implications. *Carbonates and Evaporites* 38. 10.1007/s13146-023-00909-2 (2023).

[38] Abd-Elhameed, M., Attia, G., Salama, Y., El-Moghazy, A. & Mahmoud, A. Microfacies analysis and diagenetic history of lower to middle eocene carbonates at Umm Russies area in the Northeastern desert of Egypt. *Scientific Reports* 15. 10.1038/s41598-025-05365-7 (2025). 10.1038/s41598-025-05365-7 PMC12181284 40542013

[39] Scheibner, C., Marzouk, A. M. & Kuss, J. Shelf architectures of an isolated late cretaceous carbonate platform margin, Galala mountains (Eastern Desert, Egypt). Sedimentary *Geology* 145, 23–43. 10.1016/s0037-0738(01)00114-2 (2001).

[40] Scheibner, C., Reijmer, J. J. G., Marzouk, A.M., Speijer, R. P. & Kuss, J. From platform to basin: the evolution of a paleocene carbonate margin (Eastern Desert, Egypt). *International Journal of Earth Sciences* 92, 624–640. 10.1007/s00531-003-0330-2 (2003).

[41] Kuss, J., Scheibner, C. & Gietl, R. Carbonate Platform to Basin Transition along an Upper Cretaceous to Lower Tertiary Syrian Arc Uplift, Galala Plateaus, Eastern Desert of Egypt. *GeoArabia* 5, 405–424. 10.2113/geoarabia0503405 (2001).

[42] Abdallah A. M., Sharkawi M.A., Marzouk M., 1970. The Campanian rocks of the geology of Mersa Thelmet area, Southern Galala, Plateau, A.R.E. *Bull Fac Sci Cairo Univ* 44, 271–280.

[43] Kuss, J. & U. Leppig 1989. The Early Tertiary (Middle-Late Paleocene) limestones from the western Gulf of Suez, Egypt. *Neues Jahrbuch für Geologie und Paläontologie, Monatshefte* 177, 289–332 (1989).

[44] Conoco, C. Geological Map of Egypt 1:500,000. Cairo: The Egyptian General Petroleum Corporation (1987).

[45] El Ayyat, A. M. & Obaidalla, N.A. Stratigraphy, sedimentology, and tectonic evolution of the upper Cretaceous/Paleogene succession in the North Eastern Desert, Egypt. *Journal of African Earth Sciences* 81, 35–59. 10.1016/j.jafrearsci.2013.01.007 (2013).

[46] Munsell, Soil-color chart, with genuine Munsell color chips. Grand Rapids MI 49512 (2021).

[47] Dunham, R. J. Classification of carbonate rocks according to depositional texture. - A symposium in classification of carbonate rocks, AAPG, Memoir 1: 108–121(1962).

[48] Wilson, J. L. Carbonate facies in geologic history. - Springer, Seventh Printing, 471pp (1986).

[49] Flügel, E. Microfacies of Carbonate Rocks, Analysis, Interpretation and Application. Springer, Berlin, 976p (2004).

[50] Shahar, J. The Syrian Arc system: an overview. *Palaeogeogr Palaeoclimatol Palaeoecol* 112, 125–142 (1994).

[51] Scheibner, C., Speijer, R. P., Marzouk, A. M. Turnover of larger foraminifera during the Paleocene-Eocene thermal maximum and paleoclimatic control on the evolution of platform ecosystems. *Geology* 33, 493. 10.1130/g21237.1(2005).

[52] Burchette, T. P. Tectonic control on carbonate platform facies distribution and sequence development: Miocene, Gulf of Suez. *Sedimentary Geology* 59, 179–204. 10.1016/0037-0738(88)90076-0 (1988).

[53] Hussein, I. M. & Abd-Allah, A. M. A., 2001. Tectonic evolution of the northeastern part of the African continental margin, Egypt. *Journal of African Earth Sciences* 33, 49–68. 10.1016/s0899-5362(01)90090-9 (2001).

[54] Farouk, S. Upper cretaceous sequence stratigraphy of the Galala Plateaux, Western side of the Gulf of Suez, Egypt. *Marine and Petroleum Geology* 60, 136–158. 10.1016/j.marpetgeo.2014.11.005 (2015).

[55] Kuss, J., Scheibner, C. & Gietl, R. Carbonate Platform to Basin Transition along an Upper Cretaceous to Lower Tertiary Syrian Arc Uplift, Galala Plateaus, Eastern Desert of Egypt. *GeoArabia* 5, 405–424. 10.2113/geoarabia0503405 (2000).

[56] Abdallah, A., El-Adindani, A. Note on Cenomanian-Turanian contact in the Galalah plateaus, Eastern Desert, Egypt. *Egypt J Geol* 7:171–172 (1963).

[57] Omran, A. Geophysical studies on Wadi Araba and Ras Gharib areas, Gulf of Suez, Egypt. Dissertation, Assiut University (1977).

[58] Abdel-Rahman, M., El-Etr, H. Structural pattern of the Northern part of the Eastern desert of Egypt, Apollo-Soyuz test Project, summary science report. In: El-Baz F, Warner D (eds) Vol. II: Earth Observations and Photography. NASA, Washington DC, pp 87–96 (1979).

[59] Moustafa, A.R. Structural setting and tectonic evolution of north Sinai folds, Egypt. In: Homberg C, Bachmann M (eds) Evolution of the Levant Margin and Western Arabia Platform since the Mesozoic. *Geological Society of London Special Publication* 341, pp 37–63 (2010).

[60] Yousef, M.M., Moustafa, A. R., & Shann, M. Structural setting and tectonic evolution of offshore North Sinai, Egypt. *Geological Society Special Publication* 341. 10.1144/SP341.4 (2010).

[61] Abd El-Fattah, B. K., Moustafa, A. R., & Yousef, M. A new insight into the structural evolution of Rosetta Fault, Eastern margin of Herodotus Basin, East mediterranean. *Marine and Petroleum Geology* 105161. 10.1016/j.marpetgeo.2021.105161 (2021).

[62] Bosworth, W., Lučić, D., & Stockli, D. F. North African phanerozoic. In D. Alderton & S. A. Elias (Eds.), Encyclopedia of Geology (2nd edn, pp. 244–258). Oxford: Academic Press. 10.1016/B978-0-12-409548-9.12454-6 (2020).

[63] Moustafa, A. R. Mesozoic-Cenozoic basin evolution in the northern Western Desert of Egypt. In: M. Salem, A. El-Arnauti & A. Saleh (Eds.), 3rd Symposium on the Sedimentary Basins of Libya 3, 29–46 (2008).

[64] Moustafa, A. R. Mesozoic-Cenozoic deformation history of Egypt. *Regional Geology Reviews*10.1007/978-3-030-15265-9_7 (2020).

[65] Gietl, R. Biostratigraphie und Sedimentationsmuster einer nordostägyptischen Karbonatrampe (1998).

[66] Pomoni-Papaioannou, F. Facies analysis of lofer cycles (Upper Triassic), in the Argolis Peninsula (Greece). Sedimentary Geology 208, 79–87 (2008).

[67] Davis, Jr., Dalrymple R.W. Principles of Tidal Sedimentology. Springer Dordrecht Heidelberg London New York, 621pp. DOI 10.1007/978-94-007-0123-6 (2012).

[68] Wanas, H. A. Cenomanian rocks in the Sinai Peninsula, Northeast egypt: facies analysis and sequence stratigraphy. Journal of African Earth Sciences, 52: 125–138 (2008).

[69] Mount, J. Mixed siliciclastics and carbonate sediments: a proposed first-order textural and compositional classification. - *Sedimentology* 32, 435–442 (1985).

[70] Adams, A. E. & Mackenzie, W. S. A colour atlas of carbonate sediments and rocks under the microscope. - Manson Publishing, 180pp (1998).

[71] Middleton, G. V., Church, M. J., Coniglio, M., Hardie, L. A., & Longstaffe, F. J. Encyclopedia of Sediments and Sedimentary Rocks. - Kluwer Academic Publishers; Dordrecht/ Boston/ London: 821pp (2003).

[72] Meshref, W. Tectonic Framework of Egypt. In: Said, R., Ed., Geology of Egypt, Balkema/Rotterdam/Bookfield, Netherlands, 113–156.Moustafa, A.R., Khalil, M.H., 1995. Superposed deformation in the northern Suez rift, Egypt: Relevance to hydrocarbon exploration. *Journal Petroleum Geology* 18 (3), 2455266 (1990).

[73] Schütz, K.I. Structure and stratigraphy of the Gulf of Suez, Egypt. In: Landon SM (ed) Interior rift basins, AAPG Memoir 59, pp 57–96

[74] Yousef, M.M. Structural setting of central and South egypt: an overview. *Micropaleontology* 49, 1–13 (1994).

[75] Scheibner, C. & Speijer, R.P. Late Paleocene–early eocene Tethyan carbonate platform evolution — A response to long- and short-term paleoclimatic change. *Earth-Science Reviews* 90, 71–102. 10.1016/j.earscirev.2008.07.002 (2008).

[76] Bosworth, W., & Tari, G. Hydrocarbon accumulation in basins with multiple phases of extension and inversion: examples from the Western desert (Egypt) and the Western black sea. *Solid Earth* 12, 59–77. 10.5194/se-12-59-2021 (2021).

[77] Longacre, M., Bentham, P., Hanbal, I., Cotton, J., & Edwards, R. New crustal structure of the Eastern Mediterranean Basin: detailed integration and modeling of gravity, magnetic, seismic refraction, and seismic reflection data. In EGM 2007 International Workshop: Innovation in EM, Grav and Mag Methods: A New Perspective for Exploration, Capri, Italy, 15–18 April 2007,4pp (2007).

